# Opium Use and Head and Neck Cancers: A Matched Case-Control Study in Iran

**DOI:** 10.31557/APJCP.2020.21.3.783

**Published:** 2020-03

**Authors:** Hosniyeh Alizadeh, Ahmad Naghibzadeh-Tahami, Narges Khanjani, Vahid Yazdi-Feyzabadi, Hadi Eslami, Vahidreza Borhaninejad, Mohammad Hasan Larizadeh, Ahmad Enhesari, Reza Abbasi-Rayeni, Vahid Moazed, Aliasghar Arabi Mianroodi

**Affiliations:** 1 *Neuroscience Research Center, Institute of Basic and Clinical Physiology Sciences, Kerman University of Medical Sciences, Kerman, Iran. *; 2 *Physiology Research Center, Institute of Basic and Clinical Physiology Sciences, Kerman University of Medical Sciences, Kerman, Iran. *; 3 *Environmental Health Engineering Research Center, Kerman University of Medical Sciences, Kerman, Iran. *; 4 *Adjunct Research Fellow, Monash Centre for Occupational & Environmental Health, School of Public Health and Preventive Medicine, Monash University, Melbourne, Australia. *; 5 *Health Services Management Research Center, Institute for Futures Studies in Health, Kerman University of Medical Sciences, Kerman, Iran.*; 6 *Department of Otorhinolaryngology Head and Neck Surgery, Shafa Hospital, Kerman University of Medical Sciences, Kerman, Iran. *; 7 *Social Determinants of Health Research Center, Institute for Futures Studies in Health, Kerman University of Medical Sciences, Kerman, Iran. *; 8 *Department of Radiation Oncology, School of Medicine, Kerman University of Medical Sciences, Kerman, Iran. *; 9 *Clinical Research Unit, Afzalipour Hospital, Kerman University of Medical Sciences, Kerman, Iran. *; 10 *Endocrinology and Metabolism Research Center, Institute of Basic and Clinical Physiology Sciences, Kerman University of Medical Sciences, Kerman, Iran. *; 11 *Department of Hematology and Oncology, Bahonar Hospital, Kerman University of Medical Sciences, Kerman, Iran.*

**Keywords:** Case-control, opium, risk factor, head and neck neoplasms, Iran

## Abstract

**Background:**

Head and Neck (H and N) cancers include malignant tumors of the nasal cavity, pharynx, paranasal sinuses, oral cavity, larynx and salivary glands. Opium use might be related to these cancers. The aim of this study was to investigate the relation between Opium and its Derivatives (O and D) use and the incidence of H and N cancers.

**Methods:**

In this case-control study conducted in Kerman, 140 patients with H and N cancers and 280 healthy controls (matched for age, gender, and place of residence) were included. Information about their use of O and D, cigarette smoking, alcohol and diet were collected using a structured questionnaire. Conditional logistic regression was used to investigate the relation between variables.

**Results:**

The use of opioids was associated with an increased risk of HandN cancers (Adjusted OR: 8.13; CI: 4.08-16.2). A significant dose-response relation between O and D use was observed, with high use Adjusted OR=8.91; 95% CI: 4.03-19.65 and low use Adjusted OR=6.52; 95% CI: 3.18- 13.36. This dose-response association was stronger in patients with laryngeal cancer and opioids use, with high use Adjusted OR = 11.17; 95% CI=4.48-28.09 and low use Adjusted OR = 9.46; 95% CI= 3.97- 22.52.

**Conclusion:**

The results show that opium use can be considered as an important risk factor for H and N cancers. Also in Iran, opium seems to play a more important role than cigarette smoking.

## Introduction

Head and neck (H and N) cancers are among the most frequent tumors in the world with an estimated 686,328 cases in 2012, comprising 300,373 cancers in the lip and oral cavity, 156,877 in the larynx, 142,387 in the other pharynx and 86,691 cancers in the nasopharynx. This type of cancer includes malignant tumors in the tongue, oral cavity, nasal cavity, pharynx, paranasal sinuses, larynx and salivary glands (World Health Organization, 2015). Among all H and N cancers, laryngeal cancer is the most common type and accounts for 2.2% of cancers in men and 0.4% in women and is the second most common type of respiratory tract cancers after lung cancer. In 2018, there was an estimated 650,000 cases of H and N cancers world wide and 330,000 deaths happened from this cancer (Bray et al., 2018). 

In Iran, like other parts of the world, laryngeal cancer is among the most common types of H and N cancers. But, despite the fact that H and N cancers most commonly occur in high ages in other parts of the world, these cancers including laryngeal cancer, are common under the age of 41 years in Iran (Larizadeh et al., 2014), this is probably due to different environmental exposures and risk factors in Iran (Emadzadeh et al., 2017). There is a wide geographical variation in the incidence of H and N cancers in different countries (Simard et al., 2014), which amounts to 5-50% variation across the world (Ajayi et al., 2007) . This variation may be due to differences in lifestyle, bad dietary habits, occupational exposures, vitamin deficiency, human papillomavirus (HPV) 16, and poor oral hygiene, although smoking and alcohol use are among the most common risk factors (Pezzuto et al., 2015). Some evidence shows that in some parts of the world, with decreased rates of smoking, these cancers have also decreased (Sturgis and Cinciripini, 2007). However, this decrease has not happened in other countries, which shows other important risk factors are involved as well (Blomberg et al., 2011).

On the other hand, the prevalence of alcohol consumption in some countries like Iran, is very low (Nasrollahzadeh et al., 2008; Naghibzadeh Tahami et al., 2014; Naghibzadeh-Tahami et al., 2016; Lankarani et al., 2017) and studies conducted in Iran, were not able to show a significant relation between alcohol use and other types of cancers such as esophageal (Nasrollahzadeh et al., 2008), gastric, colorectal (Naghibzadeh-Tahami et al., 2016; Lankarani et al., 2017), and bladder (Akbari et al., 2015). Therefore, other risk factors should be investigated for these cancers in Iran.

Regular use of opium maybe a risk factor for some diseases, including cancer. Previous studies have shown a relation between O and D use and the risk of laryngeal (Mousavi et al., 2003; Bakhshaee et al., 2017), gastric (Shakeri et al., 2013; Naghibzadeh Tahami et al., 2014; Sadjadi et al., 2014), esophageal (Nasrollahzadeh et al., 2008; Shakeri et al., 2012), colorectal (Naghibzadeh-Tahami et al., 2016; Lankarani et al., 2017), pancreatic (Moossavi et al., 2017) and lung (Masjedi et al., 2013)cancer. 

It was estimated that about 0.3-0.5% (12-21 billion people) around the world, aged 15-64 years, had at least a history of O and D use, in 2009 (UNODC, 2011). In Iran, 20% of the population aged 15 - 60 has a history of drug abuse, and one in 17 is a regular user(Momtazi and Rawson, 2010). In Northern Iran, about 17% of the population aged 40 and over, has a history of O and D use (Pourshams et al., 2009), and this may be one of the causes of the high incidence of esophageal cancer in this area (Nasrollahzadeh et al., 2008).

According to various studies, the prevalence of OandD use in Kerman, a city in South-East Iran, has been estimated to be between 11% and 15% (Nakhaee et al., 2008; Najafipour et al., 2015). Recently, in a study conducted on hospital in-patients and their healthy companions in Kerman, the prevalence of self-reported opium use, was 36.5% in patients and 19.3% in healthy companions (Rashidian et al., 2017). Although, there are many hospital based studies conducted on the relation between O and D use and laryngeal cancer, but as far as we know no population base study has been performed to investigate the effect O and D use on H and N cancers. 

The aim of this case-control study was to investigate the relation between O and D use and HandN cancers in a population with a relatively high prevalence of opioids use. In this study, the relation between smoking and alcohol use and the risk of HandN cancers was investigated as well.

## Materials and Methods

*Sample*

In this matched case-control study, 140 patients with HandN cancers (nasal cavity, paranasal sinuses, oral cavity, pharynx, larynx and salivary glands) whose pathologic information was registered in the Cancer Registry of Kerman University of Medical Sciences, from Jan 2014 to Dec 2017, were included. 

Sampling began by enrolling all diagnosed cases from 2017 and then enrolling cases from previous years until the required number of cases was recruited. 

Patients’ information including their home address, telephone number and cancer type were extracted from their medical records. After a telephone call to the patients and their families, a specific date and time was set up for a face-to-face interview for collecting additional information. For each case, two controls were matched for age (±5) and gender from the neighbors. In a systematic method, the nearest and first neighbors on the right side of the case home who had the inclusion criteria and willingness to cooperate in the study were selected as the matched controls. If the control was not home or did not want to cooperate, the next neighbor was approached. 

*Data Collection*

Data were collected using a two-part questionnaire. The first part consists of demographic information including gender, age, education, and marital status. The second part of the questionnaire included information about the use of OandD, alcohol, smoking and diet. The validity and reliability of this questionnaire had been approved in a previous study (Abnet et al., 2004). To minimize bias, all interviews were conducted by a trained interviewer. In order to minimize the under-reporting of opium use especially in the control group, a trained interviewer explained the purpose of the study to the participants clearly and convinced them that their information would be kept confidential. In order to maximize the participation of participants, all interviews were conducted at their homes and in a comfortable and friendly environment. 

Participant were divided into different age groups. The type and amount of opium use was questioned. In order to quantify opium and cigarette use, their amount and duration of consumption were asked. The daily use of opium was measured based on the local measurement unit, Nokhod, which is equal to 0.2 gr. The type of opioids used was divided into four categories: raw opium (teriak), sap (shireh), burned opium (sukhteh) and heroin. In this study, the use of heroin or burned opium was not reported by any subjects; therefore, only raw opium and sap entered the final analysis. 

The diet of the participants was inquired through a questionnaire which included the dietary habits of Iranians, especially local foods. 

If the person was alive, the interviews were done face-to-face, otherwise, the closest family member would be interviewed. 

*Ethics approval and consent to participate*

The aims of the study was completely explained to the participants and it was ensured that all subjects were fully aware of the scope of our study and signed an informed consent form. In this regard, ethical principles on four main issues including harm to participants; deception; invasion of privacy or lack of informed consent were considered. This study was also approved by the ethics committee of Kerman University of Medical Sciences (KUMS) with ID code KNRC/95_19/EC.

*Statistical analysis*

The cumulative consumption of each type of opioid, cigarette and alcohol was calculated as average daily use × duration of consumption, and participants were categorized into three groups; non-users, low users (≤ median use in the controls) and high users (>median use in the controls). The median use in the control group was considered as a criterion for classifying the population into high and low use groups. In all analyzes, the non-user group was considered as the reference group.

In this study, some people started opioid use after cancer diagnosis. In order to minimize reverse causality, only the history of O and D use at least 2 years before cancer diagnosis was considered. In the present study, cancers were divided into two groups; H and N cancers (nasal cavity, pharynx, paranasal sinuses, oral cavity, larynx and salivary glands), and laryngeal cancer. There were enough laryngeal cancers to do separate analysis.

In order to estimate the crude and adjusted odds ratio, conditional logistic regression with 95% confidence intervals were used. In all multivariate models, the confounding effect of only significant variables were evaluated in the univariate analysis. Moreover, only the variables with P-value less than 0.1 in the univariate analysis were added to the final multivariate models. The impact of opium exposure was calculated by adjusting the effects of other possible risk factors (cigarettes and alcohol). Multiplicative interactions between cigarette smoking (ever use) and opium use (ever use and cumulative dose) were evaluated using the likelihood ratio test. Statistical significant levels were considered P<0.05. All statistical analyses were performed using STATA 12.0 (Stata Corp, College Station, TX, USA). 

## Results

The estimated sample size for this study was174 patients and 348 controls. However, thirty four out of 174 patients did not participate in this study and the frequency of non-response was 19.5%. Therefore, 140 cases and 280 controls were included in final analysis. Most cases (79.28%) had laryngeal cancer. Among the cases, the majority (75.0%) were male and 97.14% were married. Most of the cases (64.29%) were aged 61-70 years. More than half of the cases were illiterate or only had primary school education (55.71%). Controls were well matched for age, gender; and no significant difference was observed (P>0.05). Other demographic information about cases and controls are presented in Table 1. The most common age for the onset of cigarette smoking (88.78) and opioids use (48.52%) was under 20 years and between 20-30 years. Fumigating was the most common method of use among cases (93.0%) and controls (100%). In total, 70.0% of cases and 26.79% of the controls had a history of opioids use (P<0.001).

Table 2 shows the results of opium, cigarette and alcohol use. As shown in this Table, 70% of cases with HandN cancers and 26.79% of controls had a history of opioids use. 

Based on the results, O and D use is associated with an increased risk of H and N cancers (Adjusted OR: 8.13; CI: 4.08-16.21) and laryngeal cancer (Adjusted OR: 11.98; CI: 5.05-28.39).

The amount of daily use of opium increased the risk of H and N cancers (OR: 9.22; CI: 4.19-20.28), and laryngeal cancers (Adjusted OR: 12.82; CI: 4.96-33.11). In addition, the duration of opium use, significantly increased the risk of all H and N cancers (Adjusted OR: 13.16; 95% CI: 5.32-32.5) and laryngeal cancer (Adjusted OR: 13.68; 95% CI: 5.12-36.56). 

There was a dose-response relationship between cumulative use of opium and the incidence of all H and N cancers (Adjusted OR: 8.91; 95% CI: 4.03-19.65). The dose-response relationship was also observed in laryngeal cancer (Adjusted OR: 11.17; 95% CI: 4.44-28.09).

About 65% of cases were smokers, while this rate was 40% for controls. In univariate analysis, we observed a 5-time increase in H and N cancer risk associated with smoking tobacco.(OR= 4.9; 95% CI: 2.65-9.04), but also multivariate analysis showed that after adjustment of diet and education, the relationship was significant (Adjusted OR: 3.23; 95% CI: 1.69-6.17). The odds ratio was significant in laryngeal cancer (Adjusted OR: 4.14; 95% CI: 1.93-8.87) as well. The association was hold when we examined duration and intensity of tobacco smoking. Daily use of cigarettes had a significant relation with HandN cancers (Adjusted OR: 4.41; 95% CI: 1.78-7.58). Multivariate analysis also showed that the daily use of cigarettes was associated with an increased risk of laryngeal cancer (Adjusted OR: 5.41; 95% CI: 2.24-13.22). Similarly, the duration of cigarette smoking had a statistically significant association with the risk of H and N cancers (Adjusted OR: 4.13; 95% CI: 1.83-9.30) and laryngeal cancer (Adjusted OR: 5.76; 95% CI: 2.27-14.59).

Multivariate analysis showed a significant association between the cumulative use of cigarettes and HandN cancers (OR: 4.06; 95% CI: 1.90-8.67) and laryngeal cancer (OR: 5.16; 95% CI: 2.19-12.16). 

The interaction between cigarette use (ever use) and opium use (ever or cumulative dose) was not statistically significant in HandN cancers (P=0.60 and 0.76, respectively) or laryngeal cancers (P=0.13 and 0.35, respectively).

In H and N cancers, 10% of cases and 4.29% of controls had a history of alcohol use. In this group, the odds ratio was significant in multivariate analysis (Adjusted OR= 4.26; 95% CI: 1.67-10.84). Similarly, in the laryngeal cancer group, after adjustment for other confounding factors, alcohol use was associated with an increased risk of laryngeal cancer (Adjusted OR: 5.00; 95% CI: 1.72-14.48). 

Also, a significant relation was observed in multivariate analysis between daily alcohol use and HandN cancers (Adjusted OR: 7.36; 95% CI: 1.38- 29.32). Similarly, there was a significant association between daily alcohol use and laryngeal cancer (Adjusted OR: 8.85; 95% CI: 2.64-30.07).

Also, after adjustment for confounders, duration of alcohol use was associated with HandN cancers (Adjusted OR: 6.56; 95% CI: 2. 47-28.34) and laryngeal cancer (Adjusted OR: 7.36; 95 % CI: 3.48-27.32). The cumulative use of alcohol, was associated with H and N cancers (Adjusted OR: 6.36; 95% CI: 2.38-29.32) and laryngeal cancers (Adjusted OR: 7.19; 95% CI: 2.79-32.87).

**Table 1:** Demographic Variables of Case and Control Groups

Variable	All Head and Neck cancers †	Matched controls	Laryngeal cancer † †	Matched controls
N	140	280	111	222
Gender				
Male	105 (75.0)	210 (74.2)	95 (85.59)	190 (85.59)
Female	35 (25.0)	70 (25. 0)	16 (14.41)	16 (14.41)
P-value	1.0		1.0	
Marital status				
Married	136 (97.14)	267 (95.36)	109 (98.20)	215 (96.85)
Single	4 (2.86)	13 (4.64)	2 (1.8)	7 (3.15)
P-value*	0.38		0.47	
Age				
≤ 50	35 (25.0)	69 (24.64)	24 (21.62)	48 (21.62)
51-70	90 (64.29)	179 (63.93)	76 (68.47)	150 (67.57)
> 70	15 (10.71)	32 (11.43)	11 (9.91)	24 (10.81)
P-value*	0.97		0.96	
Education				
Illiterate and elementary	78 (55.72)	73 (26.07)	66 (59.46)	59 (26.58)
Middle and high school	50 (35.72)	117 (41.79)	38 (34.23)	92 (41.44)
High School Diploma and above	12 (8.58)	90 (32.14)	7 (6.31)	71 (31.98)
P-value*	0.001		0.001	

† Includes nasal cavity, pharynx, paranasal sinuses, oral cavity, larynx, and salivary glands cancers. †† Includes only laryngeal cancer; *P value calculated using McNemar’s test.

**Table 2:** The Association between the Use of Opioid and Its Derivatives, Cigarette, Alcohol and the Incidence of H and N Cancers

Variable	All Head and Neck cancers				Laryngeal cancer			
Opium	Cases N (%)	Controls N (%)	Unadjusted OR (95%CI)	Adjusted † OR (95%CI)	Cases N (%)	Controls N (%)	Unadjusted OR (95%CI)	Adjusted † OR (95%CI)
Opium use								
Never	42 (30.00)	205 (73.21)	Referent	Referent	23 (20.72)	158 (71.17)	Reference	Reference
Ever	98 (70.00)	75 (26.79)	11.82 (6.07- 23.0)	8.13 (4.08- 16.21)	88 (79.28)	64 (28.83)	17.52 (7.56- 40.60)	11.98 (5.05-28.39)
Amount of daily opium use								
Never used	42 (30.00)	205 (73.21)	Referent	Referent	23(20.72)	158 (71.17)	Reference	Reference
≤Median ††	45 (32.14)	43 (15.36)	9.42 (4.55- 19.47)	7.19 (3.32- 15.60)	41 (36.94)	36 (16.22)	14.67 (5.95-36.20)	11.17 (4.33- 28.83)
>Median	53 (37.86)	32 (11.43)	15.30 (7.12- 32.93)	9.22 (4.19- 20.28)	47 (42.34)	28 (12.61)	21.07 (8.35-53.13)	12.82 (4.96- 33.11)
Duration								
Never used	42 (30.00)	205 (73.21)	Referent	Referent	23 (20.72)	158 (71.17)	Reference	Reference
≤Median	58 (41.43)	47 (16.79)	8.37 (4.43- 15.81)	5.65 (2.90- 10.98)	57 (51.35)	40 (18.02)	13.96 (6.23- 31.27)	7.05 (3.17- 15.67)
>Median	40 (28.57)	28 (10.00)	19.09 (7.87-46.25)	13.16 (5.32-32.53)	31 (27.93)	24 (10.81)	19.79 (7.28- 53.78)	13.68 (5.12- 36.56)
Cumulative use of Opium†††								
Never used	42 (30.00)	205 (73.21)	Referent	Referent	23 (20.72)	158 (71.17)	Reference	Reference
≤Median††	48 (34.29)	42 (15.00)	9.05 (4.60-18.05)	6.52 (3.18- 13.36)	44 (39.64)	35 (15.77)	13.23 (5.73-30.53)	9.46 (3.97- 22.52)
>Median	50 (35.71)	33 (11.79)	14.07 (6.59-30.03)	8.91 (4.03- 19.65)	44 (39.64)	29 (13.06)	18.40 (7.54-44.90)	11.17 (4.44- 28.09)
Cigarette smoking								
Never	48 (34.29)	167 (59.64)	Referent	Referent	26 (23.42)	119 (53.60)	Reference	Reference
Ever	92 (65.71)	113 (40.36)	4.90 (2.65- 9.04)	3.23 (1.69- 6.17)	85 (76.58)	103 (46.40)	6.28 (3.06-12.88)	4.14 (1.93- 8.87)
Amount of daily tobacco use								
Never used	48 (34.29)	167 (59.64)	Referent	Referent	26 (23.42)	119 (53.60)	Reference	Reference
≤Median ††	33 (23.57)	57 (20.36)	3.18 (1.58-6.39)	2.54 (1.34-6.26)	29 (26.13)	51 (22.97)	3.92 (1.74-8.81)	3.42 (1.56-6.67)
>Median	59 (42.14)	56 (20.00)	7.41 (3.65-15.04)	4.41 (1.78-7.58)	56 (50.45)	52 (23.42)	9.20 (4.14-20.47)	5.41 (2. 24-13.22)
Duration								
Never used	48 (34.29)	167 (59.64)	Referent	Referent	26 (23.42)	119 (53.60)	Reference	Reference
≤Median ††	54 (38.57)	78 (27.86)	4.07 (2.14- 7.73)	2.90 (1.46- 5.73)	49 (44.14)	71 (31.98)	5.08 (2.40- 10.73)	3.58 (1.61- 7.94)
>Median	38 (27.14)	35 (12.50)	7.46 (3.49- 2.39)	4.13 (1.83- 9.30)	36 (32.43)	32 (14.41)	9.98 (4.19-23.74)	5.76 (2.27- 14.59)
Cumulative use of cigarette smoking †††								
Never used	48 (34.29)	167 (59.64)	Referent	Referent	26 (23.42)	119 (53.60)	Reference	Reference
≤Median††	37 (26.43)	57 (20.36)	3.65 (1.83- 7.27)	2.67 (1.29- 5.50)	33 (29.73)	51 (22.97)	4.61(2.07-10.27)	3.36 (1.44-7.81)
> Median	55 (39.29)	56 (20.00)	6.59 (3.26-13.30)	4.06 (1.90- 8.67)	52 (46.85)	52 (23.42)	8.20 (3.71-18.13)	5.16 (2.19-12.16)
Alcohol use								
Never use	126 (90.00)	268 (95.71)	Referent	Referent	100 (90.09)	212 (95.50)	Reference	Reference
Ever use	14 (10.00)	12 (4.29)	2.54 (1.12- 5.79)	4.26 (1.67- 10.84)	11 (9.91)	10 (4.50)	2.43 (0. 96 - 6.14)	5.00 (1.72-14.48)
Amount of daily Alcohol use								
Never used	126 (90.00)	268 (95.71)	Referent	Referent	100 (90.09)	212 (95.50)	Reference	Reference
≤Median††	5 (3.57)	6 (2.14)	1.78 (0. 53- 5.89)	1.83 (0. 41-6.91)	4 (3.60)	6 (2.70)	1.50 (0. 38-5.84)	0.85 (0. 14-4.84)
> Median	9 (6.43)	6 (2.14)	3.42 (1.13- 10.34)	7.36 (1. 38- 29.32)	7 (6.31)	4 (1.80)	3.57 (1. 04-12.24)	8.85 (2. 64-30.07)
Duration								
Never used	126 (90)	268 (95.71)	Referent	Referent	100 (95.50)	212 (95.50)	Reference	Reference
≤Median††	5 (3.57)	6 (2.14)	1.89 (0. 56- 6.30)	1.62 (0. 50- 5.22)	3 (2.7)	5 (2.25)	1.42 (0. 33-6.14)	1.73 (0. 71-8.22)
> Median	9 (6.43)	6 (2.14)	3.15 (1.11- 8.93)	6.56 (2. 47- 28.34)	8 (7.21)	5 (2.25)	3.29 (1.06-10.17)	7.36 (3.48-27.32)
Cumulative use of alcohol use †††								
Never used	126 (90)	268 (95.71)	Referent	Referent	100 (90.09)	212 (95.50)	Reference	Reference
≤Median	5 (3.57)	7 (2.50)	1.62 (0. 50- 5.22)	1.93 (0. 51- 7.22)	3 (2.70)	6 (2.70)	1.19 (0. 29- 4.90)	1.84 (0. 37- 9.15)
> Median	9 (6.43)	5 (1.79)	3.74 (1.24- 11.25)	6.36 (2.38- 29.32)	8 (7.21)	4 (1.80)	4.06 (1.21-13.59)	7.19 (2. 79-32.87)

† Confounding effect of specific dietary factors such as the use of meat, fruit and vegetables, hydrogenated fats, olive oil as well as levels of education were controlled. †† Median of use in the control group was considered as a criterion. †††Cumulative use was obtained by multiplying the amount of use (per day) and the duration of use (per year).

## Discussion

The present study is probably the first case-control study that showed a dose-response relation between opium use and HandN cancers, although the relation between opium use and laryngeal cancer has been investigated in previous studies (Mousavi et al., 2003; Bakhshaee et al., 2017), in none of these studies, was the dose-response relationship evaluated.

The results of this study showed that the majority of the patients with H and N cancer had a history of opium use, and use of O and D may be associated with an increased risk of H and N cancers. Before causal inference, methodological problems should be considered. The association between opium use and an increased risk of cancers may be confounded by other risk factors such as age, gender, use of cigarette and alcohol (Naghibzadeh Tahami et al., 2014). Opium consumers are elderly people who are more likely to be cigarette smokers; however, in this study after adjustment for confounders, including cigarette smoking, alcohol use and diet, the results remained significant. 

Evidence provided by this study intensifies the existence of a causal relation between opium use and H and N cancers, as the relation observed in this study was relatively strong and opium use increased the risk of H and N cancers and laryngeal cancer over 12 and about 17 times respectively. Other studies conducted in Iran have shown a relation between O and D use and laryngeal cancer as well. In a hospital-based study conducted in Kerman, opium use increased the risk of laryngeal cancer over 10 times (95% CI: 5.76–20.0) (Mousavi et al., 2003).Another study conducted in Tehran, Iran, also showed that after controlling the effect of smoking, O and D use increased the risk of laryngeal cancer about 6 times (95% CI:1.10-33.23) (Bakhshaee et al., 2017). In a recent study, in Israel a significant relation was observed between IV (intravenous) O and D use and Supraglottic Squamous Cell Carcinoma and the odds ratio for patients with an IVDA history to have SG-SCC relative to G-SCC was 10.84 (95% CI: 1.3-89.4) (Shoffel-Havakuk et al., 2017). Another study showed the odd ratio between opium use and the risk of oral cancers (tongue, palate, and buccal), to be 4.0 (95% CI: 1.2-13.6) (Razmpa et al., 2014). Also several studies showed that OandD use may increase the risk of cancers such as gastric (Shakeri et al., 2013; Naghibzadeh Tahami et al., 2014), esophageal (Hewer et al., 1978; Nasrollahzadeh et al., 2008; Shakeri et al., 2012), bladder (Akbari et al., 2015), lung(Masjedi et al., 2013), colorectal (Naghibzadeh-Tahami et al., 2016; Lankarani et al., 2017), and pancreatic (Moossavi et al., 2017).Consistent with this study, a dose-response relationship was observed in most of these studies (Nasrollahzadeh et al., 2008; Lambert et al., 2011; Naghibzadeh Tahami et al., 2014; Naghibzadeh-Tahami et al., 2016; Moossavi et al., 2017). However, some studies have not mentioned this relationship (Mousavi et al., 2003; Bakhshaee et al., 2017).

The consistency of the results in various studies may confirm the casual association reported in the present study and the carcinogenic effects of opium. Since opioids may be used to relieve cancer pain, in order to prevent reverse causality, the history of O and D use from at least 2 years before the date of cancer diagnosis was investigated and this represents the temporal relationship of association.

Many mechanisms have been proposed regarding the relation between opium use and the incidence of cancer. The use of opium and its alkaloids, including morphine may have mutagenic effects (Friesen et al., 1985). Empirical studies have shown that opium pyrolysis has mutagenic effects on Salmonella strains (Nasrollahzadeh et al., 2008). Also pyrolysis and alkaloids of morphine lead to sister chromatic exchange in human lymphocytes and morphological changes in cultured Syrian hamster embryo cells (Perry et al., 1983). These drugs have caused carcinogenic changes after being injected into tissues, under the skin, inside the trachea and into the gastrointestinal system of hamsters (Friesen et al., 1985). It has also been shown that pyrolysis and aromatic hydrocarbons (PAHs) released from burning opioid substances indirectly lead to DNA damage and, as a result, may stimulate mutagenic mechanisms (Nasrollahzadeh et al., 2008). In general, the carcinogenic mechanisms of opium have not been well identified and further studies are required to understand the carcinogenic mechanism of opium. H and N cancers are multifactorial and while opium is a rather prevalent and potent risk factor but it is possible that other factors (such as HPV) would have less prevalence in this population.

In addition, many impurities are added to opioids during their processing in Iran and these chemicals may have carcinogenic effects. One of these chemicals is lead, which is added in order to make it heavier so that drug dealers can make more profit. In studies conducted on addicted people in Kerman, the amount of lead in the blood of these people was higher than usual. The non-organic lead in these drugs can cause severe toxic and even carcinogenic effects (Hayatbakhsh et al., 2017). The relation between occupational exposure to lead and gastric, lung and thyroid cancers have been shown in several studies (Wong and Harris, 2000). 

While smoking and alcohol use are two main risk factors for HandN cancers (Malhotra et al., 2017), in this study, the association between smoking and the incidence of H and N cancers was also statistically significant. In Iran, since almost all people are Muslims, the prevalence of alcohol use is much lower than in other parts of the world (Mozafarinia et al., 2017). 

Our study showed a relatively strong association between cigarette smoking and HandN and laryngeal cancers in univariate analysis which is consistent with many other studies in Iran and across the world (Jethwa and Khariwala, 2017; Smith et al., 2019). However, after adjustment for other confounding factors, cigarette smoking significantly increased the risk of H and N and laryngeal cancers. Although the results are similar to other countries across the world, but the strength of this relationship in Iran is weaker (Mousavi et al., 2003; Malhotra et al., 2017). The reason for these different results are not clear to us, although this could be due to some reasons such as the lower intensity or lower cumulative use of cigarettes in Iran or the different type of tobacco (Shakeri et al., 2013). Some other studies conducted in Iran showed no relation between smoking and esophagus (Nasrollahzadeh et al., 2008), colorectal (Naghibzadeh-Tahami et al., 2016; Lankarani et al., 2017), gastric (Shakeri et al., 2013; Naghibzadeh Tahami et al., 2014) and bladder(Akbari et al., 2015)cancers. Also, two studies conducted in Linxian (China) and Golestan (Iran), which are areas with a high risk of esophageal cancer, showed a weak to moderate association between smoking and esophageal cancer, and authors concluded that smoking was not a strong risk factor for esophageal cancer in these specific populations (Tran et al., 2005). This might indicate that there are other stronger risk factors for H and N cancers and one might be opium.

Opioids use has a high prevalence in some parts of Iran. A study conducted in Golestan province showed that 17% of adults had a history of opioids use (Sadjadi et al., 2014), and based on current evidence, Kerman province is considered as one of the areas with a relatively high prevalence of opium use (Nakhaee et al., 2008; Najafipour et al., 2015; Rashidian et al., 2017). Based on this information and the existence of a strong relation between opium use and cancers, as well as high use of opium in some areas, a significant proportion of the burden of these cancers may be related to opioids use. It is estimated that there are about 15 million opioids consumers in the world (Shakeri et al., 2013), which is a great concern. 

*Study strengths and limitations*

This study had some limitations. One of the limitations was its low sample size, therefore different HandN cancers could not be analyzed separately. In addition, this study like other case-control studies was prone to recall bias, interviewer, and reporting bias. Under-reporting of opium use, especially in controls, was another possible bias, although by standardizing the interview process and employing a trained interviewer to conduct all interviews, the possible effect of this bias was minimized. Another limitation of this study is the wide confidence interval that may be due to the low sample size.

The most important strength of this study was that all cancer cases included had been confirmed pathologically, which minimized the probability of misclassification bias. Another strength of this study was using population-based neighbor controls rather than hospital controls which led to better control over socio-economic factors (Shakeri et al., 2012). This study used a questionnaire which its validity and reliability have been proven in similar previous studies (Abnet et al., 2004).

Moreover further studies, such as prospective studies, may be helpful in further proving this casual relation, although it seems worthwhile to state that most studies on the association between opium and cancer are from Iran.

In conclusion, this study showed that O and D use may be a risk factor for H and N cancers, including laryngeal cancer in Iran. There was a dose-response association. There was another dose-response relation that was related to cigarette smoking, however, there was no multiplicative interaction between cigarette smoking and opium use. On the other hand, due to the low sample size, the results should be interpreted with caution. The results are consistent with previous literature showing that opium is related to cancer.

*Abbreviations*

CI: Confidence interval; H and N: Head and Neck; O and D: Opium and its Derivatives; OR: Odds ratio.
